# Large Male Caterpillars Are the Primary Builders: Exploring Tent Construction and Foraging Behaviour in Gregarious Pine Processionary Caterpillar

**DOI:** 10.3390/insects14100829

**Published:** 2023-10-21

**Authors:** Mizuki Uemura, Myron P. Zalucki, Andrea Battisti

**Affiliations:** 1 School of the Environment, The University of Queensland, St Lucia, QLD 4072, Australia; 2 Department of Agronomy, Food, Natural Resources, Animals and Environment, University of Padova, 35020 Legnaro, Italy; andrea.battisti@unipd.it

**Keywords:** colony, individual differences, pine processionary moth, *Thaumetopoea pityocampa*, Lepidoptera, Notodontidae

## Abstract

**Simple Summary:**

*Thaumetopoea pityocampa* caterpillars are a significant pest of conifer trees in Europe and live gregariously in a communal tent with siblings and conspecifics from other colonies. Despite their economic and medical importance, there has been a lack of quantitative information on the tent construction and foraging behaviour in *T. pityocampa* colonies. In this study, we observed the tent construction and foraging behaviour of *T. pityocampa* caterpillars in the field at Tregnago, Verona, Italy. At around sunset, large male caterpillars emerged from the tent first to construct the tent, while many female caterpillars emerged later in the night only to forage. As younger instar caterpillars moulted to older larval instars, the environmental temperature decreased, which consequently increased the duration of their foraging activities. The final tent structure constructed by later instar caterpillars was not isodiametric; more silk was applied on the southern side of the tent to receive maximum insolation during the winter months. This study demonstrated the importance of the winter tent and individual variation in tent construction and foraging behaviour of *T. pityocampa* caterpillars amongst sex, body size, and larval instar and lays the basis for further investigation in polyethism.

**Abstract:**

As a social organism, living in a communal structure is one of the most important physical barriers against environmental elements and natural enemies. *Thaumetopoea pityocampa* (Notodontidae, Thaumetopoeinae) caterpillars are conifer pests that spend most of their larval stage in winter. Although *T. pityocampa* holds economic and medical significance, the tent construction and foraging behaviour are poorly understood. We observed the tent construction behaviour in autumn (October and November) when third- and fourth-instar *T. pityocampa* caterpillars build the ‘winter tent’ that can withstand winter conditions. Just before sunset, with no rain and temperatures over 12 °C, tent construction was undertaken by early active individuals, primarily larger male caterpillars. Early active caterpillars emerge from the tent first and spin silk on the tent for expansion and strength. Once temperatures dropped below 12 °C and twilight had passed, the early active caterpillars went out to forage and were later joined by the late active caterpillars, which were predominantly smaller females that had remained inside the tent. Foraging behaviour was continuously monitored for the first to fourth larval instars in the field. Foraging was more frequent in younger instars when environmental temperatures were warmer and became continuous and prolonged in later instar caterpillars as temperatures dropped. The final tent structure built by later instar caterpillars had the thickest layer of silk on the southern side of the tent compared to other orientations to receive maximum solar radiation during the winter. Our study provided additional insights into the collective nest building, foraging and social behaviours observed in Lepidoptera, as well as the roles of individuals within non-eusocial insect colonies.

## 1. Introduction

Many social insects rely on a nesting structure where they can be protected from environmental elements and natural enemies and/or benefit from a modified microclimate [1]. Amongst social insects, the most intricate and well-known nesting structures are those made by eusocial insects from Hymenoptera (ants, bees and wasps) and Blattodea (termites) [2]. Naturalists and scientists have had curiosities about the behaviour, mechanisms and purpose of these structures in various groups of insects. Excluding Hymenoptera and Blattodea, Lepidoptera are the only insects to exhibit true collective nest-building behaviour that is synchronised within the colony [3]. Yet the behavioural mechanisms behind nesting structures of gregarious Lepidoptera are one of the least studied amongst social insects [3]. Nests built by caterpillars are formed either by pulling together plant parts with silk or by spinning silk on the framework of branches and leaves [3]. Studies on nest-building behaviour have been conducted on species from Erebidae [4], Lasiocampidae [5,6,7], Notodontidae [8,9], Pieridae [10,11], Tortricidae and Yponomeutidae [3]. 

The communal silken structure (tent) forms a vital part of the pine processionary moth *Thaumetopoea pityocampa* (Denis and Schiffermüller) (Notodontidae: Thaumetopoeinae) lifecycle and larval development. *Thaumetopoea pityocampa* caterpillars are gregarious, with five larval instars. The caterpillars build several temporary tents as younger larval instars at the end of summer/early autumn, and in autumn, the later larval instars build a thick-walled permanent ‘winter tent’, which is positioned towards the top of the host tree [12]. *Thaumetopoea pityocampa* is a widespread pest of conifer trees in the Mediterranean basin and Southern Europe [13] that also causes medical complications in humans and domestic animals [14]. One of the key traits contributing to the widespread colonisation of this species is the ability to endure winter conditions by living in a communal tent. Yet, no quantitative experiments have been conducted to investigate this tent-constructing behaviour. 

The first description of *T. pityocampa* and the tent structure was written by [15], which was followed by [9,16]. The thermal functionality of the *T. pityocampa* tent was described by [9], which was shown quantitatively by [17,18]. Studies by [9] observed that as soon as night falls, the caterpillars become active, and each individual belongs to one of three groups: (I) First group of caterpillars distribute on the tent surface and enlarge the tent by adding silk to the structure before foraging; (II) Second group of caterpillars remain just inside the outer tent and fix loose silk strands within, which is then followed by foraging, and (III) Last group go out to forage and do not undertake spinning. The enlargement of the tent through spinning is prompted by shorter day length and a drop in night temperatures [9]. 

In this study, we wanted to conduct detailed observations and quantitative analyses of the social behaviours associated with tent construction and foraging in *T. pityocampa* caterpillars. *Thaumetopoea pityocampa* caterpillars spend their life in a tent with their siblings and conspecifics from other colonies. In a species with such gregarious behaviour from egg to moth [19], it is expected that individuals have cooperative and synchronised activities to perform well as a colony. Tent construction is directly followed by foraging. Therefore, in this study, we quantitatively investigated tent construction and foraging behaviour across first (L1) to fourth (L4) larval instar *T. pityocampa* colonies. We used a novel frass-collecting apparatus and wildlife cameras, allowing us to document the natural behaviours of *T. pityocampa* colonies in the field over 24 h periods.

## 2. Materials and Methods

### 2.1. Study Organism

In summer, each female *T. pityocampa* moth oviposits all her eggs in one batch (150–350 eggs) on the outer foliage of a pine tree, and the eggs hatch in a month [13]. The species has a univoltine lifecycle and is gregarious from egg to moth [19]. Unlike most defoliating insects of temperate regions, the caterpillars feed throughout the winter. Neonates build their first temporary tent at the oviposition site, and the second larval instar generally builds a new temporary tent on the same branch as the first [12]. Later larval instars (L3–4) construct a thick-walled permanent tent (winter tent) at the outermost parts of the tree in more sun-exposed locations [12]. From approximately L3 onwards, caterpillars exclusively forage at night and return to the tent before sunrise. During the coldest months (December to February), they leave the tent for feeding only if the internal tent temperature achieves 9 °C during the day and is above 0 °C at night [17]. During winter, the tent facilitates heating the body temperature of caterpillars during the daytime [18], which is fundamental for stimulating feeding at night [17]. Additionally, the tent acts as protection against predators, parasitoids and environmental elements such as wind, rain, and snow [20,21]. When final instar caterpillars are ready to pupate, they leave the tent and crawl in a procession on the ground to search for a pupation site, which is usually in brightly lit areas of the environment [22].

### 2.2. Daily Caterpillar Activity: Frass Production

Preliminary experiments on *T. pityocampa* frass collection were tested at an indoor laboratory before experimenting in the field. From mid-August to the end of September 2020, when *T. pityocampa* caterpillars are L1 to L2, frass production of 12 colonies in the field (Precastio Tregnago Verona Italy, 45°31′ N, 11°10′ E, 530 m; Supplementary Figure S1) were measured and analysed. The larval instar was determined by the body and frass size according to Dyar’s law. Caterpillars that are L3 and older feed beyond the branches surrounding the tent [12]; therefore, reliable frass counts could not be recorded for older instar caterpillars. We randomly selected ten L1–2 colony tents that were a maximum of 1 m off the ground on *Pinus nigra* host trees. The frass counts were performed using a frass collecting apparatus made from a rotating disc placed directly underneath the tent where younger instar caterpillars feed in the immediate area [12]. The rotating disc was built by attaching a 21 cm diameter circular corrugated sheet on a 24 h mechanical plug-in timer using Scotch clear extreme fastener strips. A transparent circular sheet of plastic printed with a circle containing 24 equal slices was placed on top of the corrugated sheet. The disc rotates with the 24 h mechanical plug-in timer, and each slice represents one hour of the day. Coloured paperclips were used as markers to determine the tent’s position relative to the disc. In the field, the wind caused the frass to move; therefore, the plastic sheet was coated with liquid Vaseline to keep the frass in place. The rotating disc was attached to a metal ring of an 80 cm steel stand with heavy-duty tape (Supplementary Figure S2A,B). The timers were powered using the Wolf PRO Power Bank (Litionite, Ancona, Italy), and the power bank was sheltered from the rain and wind by an umbrella tied to the tree branches. One rotating disc was placed under each colony, and two colonies were surveyed for 24 h. No more than two discs could operate at one time because of the power bank battery capacity. The same colony was not used to measure the frass of more than one instar. The experiment generally started in the afternoon when no larval activity was observed and stopped after approximately 24 h. A photo of the disc was then taken from above using an iPhone (Supplementary Figure S2C), and the disc was cleaned and recoated with liquid Vaseline for the next frass collection. The images were cropped to each slice, and the frass on each slice was digitally counted using ImageJ version 1.53e with the Analyze Particles function (Supplementary Function S1).

### 2.3. Daily Caterpillar Activity: Tent Construction and Foraging

Tent construction behaviour of 45 *T. pityocampa* colonies of L1 to L4 larval instar were monitored in the field from July 2020 to February 2021. Final instar L5 caterpillars were not included in the experiment because they are less involved in tent construction [23]; this was also confirmed by observations carried out by one of the authors (Battisti, pers. obs.) in the same site in previous years. Colonies were randomly selected from ten isolated *P. nigra* host trees. Protective gear against urticating setae was worn when handling colonies to minimise the experimenter’s exposure. 

From mid-August to the start of November 2020, when caterpillars were L2–L4, two wildlife cameras were set up on a tripod and focused on taking images of a single colony each every 15 min for 24 h. Every two to three days, the cameras were relocated to capture images of different colonies and the same colony was not used again. The images were stored on an SD card and reviewed on a computer. The timestamp and larval activity captured on each image, including spinning silk/tent construction (spinning), feeding and returning to the tent (foraging), were noted for each colony. Spinning activity was identified when caterpillars were visible through the silk strands of the tent and when seen moving on the tent surface. The foraging activity was identified when caterpillars moved in a procession from the tent to nearby pine needles and engaged in feeding on the pine needle. Caterpillars returning to the tent were identified when caterpillars moved in a procession from the pine needles back to the tent and remained inside. Individual caterpillars could not be distinguished through the wildlife camera images; therefore, the activities were analysed collectively as a colony and not attributed to specific individuals. To further validate the larval activity times from the images, the colonies were observed daily in the field at different times of the day and night. When conducting observations of *T. pityocampa* colonies at night, a red-light headlamp was used to minimise disturbance. Many colonies of L4 caterpillars built their tent higher in the tree than the height of the tripod [12]; therefore, images could only be taken for those colonies that were accessible. HOBO Temperature/RH data logger (Onset Computer Corporation, Bourne, MA, USA) probes were placed next to the monitored tent to measure the environmental temperature every 15 min.

In October-November 2020, when caterpillars were L3 and spun the most amount of silk for tent construction (Supplementary Figure S3), we analysed the wildlife camera images of eight colonies. Due to the large size of the individuals, we were able to distinguish the spinners and the foragers clearly in the images. For each activity type (spinning and foraging), the time, environmental temperature, average daily temperature, colony identification (ID), and weather conditions were recorded. Additionally, we noted the wildlife camera orientation relative to each colony.

In January, when *T. pityocampa* is L5, the caterpillars reduce their daily tent construction activities, and the winter tent is in its completed form (Uemura, pers. obs.). The spiral characteristics (phyllotaxis) of *P. nigra* needles on the shoots provide a consistent spherical platform for silk attachment/tent construction for *T. pityocampa* caterpillars. To determine if the final tent structure is symmetrical and has the same diameter all around (isodiametric), we measured the radii of 100 winter tents built by later instar larvae from Monte Garzon, Italy (45°31′ N, 11°10′ E) in January 2021. The pine shoot was used as the central reference point, and the radii were measured with a ruler going outwards towards N, E, S and W. The four cardinal orientations of the tent radii were distinguished by using the iPhone application Compass. The tent measurements were conducted from the ground or by using a ladder or tree-climbing equipment.

### 2.4. Polyethism

We observed that not all caterpillars come out of the tent at sunset to spin silk to enlarge and construct the tent. We hypothesised that there could be polyethism in the colonies, meaning that specific individuals have specialised roles and responsibilities. During our observations from wildlife camera images and field visits at sunset and in the evening, we were able to identify two distinct caterpillar activities in all *T. pityocampa* colonies. (1) At sunset, the early active caterpillars spun silk on the tent and foraged immediately after the spinning activity ceased. (2) Meanwhile, the late active caterpillars emerged from the tent and foraged from around midnight to early morning.

Across two nights in November to December 2020 and five nights in November to December 2021, we collected 16 and 25 L4 caterpillar tents with pine branches, respectively, from Precastio. The tents were transported to a nearby outdoor facility and individually hung across a 6 m clothesline for monitoring caterpillar activity throughout the night and early morning. We collected 10 to 30 early and late active caterpillars with forceps from 20 (2020: *N* = 10, 2021: *N* = 10) and 21 (2020: *N* = 6, 2021: *N* = 15) tents, respectively, and stored them in 70% ethanol. Protective clothing and eyewear were worn during the collection to eliminate contact with urticating setae from the caterpillars. From the onset of darkness, we used a red headlamp to determine and differentiate the behaviour of the caterpillars without disturbing their behaviour. Differentiating sex using external morphology [24,25] was not applicable for *T. pityocampa* caterpillars. Therefore, collected caterpillars were dissected, and the presence or absence of the testis located dorsally on the fifth abdominal segment was used to determine if the caterpillar was male or female [26]. We measured the head capsule width of dissected caterpillars using a Leica S9i Digital Stereo Microscope at 20× magnification with an eyepiece graticule. 

### 2.5. Statistical Analyses

The following statistical analyses were performed using R Studio version 4.1.0 (R Core Team, Vienna, Austria) and GraphPad Prism 9 (GraphPad Software, Boston, MA, USA). An alpha value of *p* < 0.05 was taken as statistically significant.

#### 2.5.1. Daily Caterpillar Activity: Frass Production

The quantity of frass produced by L1–L2 *T. pityocampa* colonies was counted for each slice (total of 24 slices) from a disc, which represented an hour within that day. Since the frass production varies across colonies due to differences in the sizes/number of caterpillars in each colony, the data was standardised. The frass count for each hour/slice of the day was divided by the total frass count from the same 24 h disc. The standardised hourly frass count was then averaged across all colonies within a larval instar. The frass data was plotted with the average foraging activity from the wildlife camera (see below) using GraphPad Prism 9 (GraphPad Software, Boston, MA, USA). For the graphical presentation, the standardised frass count was multiplied by 10 to compare it with the average foraging activity easily. 

#### 2.5.2. Daily Caterpillar Activity: Tent Construction and Foraging

The time at which *T. pityocampa* caterpillars went out to spin silk, forage and return to the tent was noted for each colony by visually analysing the wildlife camera images. The time of activity was converted to frequency by averaging the counts of activity per hour from all colonies within a larval instar. The frequency of activity ranged from 0 to 1, where 0 indicated no active colonies, and 1 indicated that all colonies within the larval instar were active at that time. Hourly temperatures were calculated by averaging environmental temperatures collected from data loggers during the developmental period that each larval instar experienced. Hours of darkness were calculated by taking the average of the sunrise and sunset times retrieved from [27] for the developmental period experienced by each larval instar. The data (L1: *N* = 14; L2: *N* = 11; L3: *N* = 15; L4: *N* = 5 colonies) was plotted using GraphPad Prism version 9.0.0 for Windows. 

An analysis of tent construction behaviour was conducted on L3 *T. pityocampa* colonies when the caterpillars spun the most silk on the tent. Tent construction behaviour in L3 caterpillars was analysed using a generalised linear model (GLM) to determine if the time of the two activities (spinning and foraging) was associated with various independent variables. To take visible light into account, the time at sunset must be considered because it differs day by day. Therefore, the time of activity was standardised to minutes before and after sunset. The sunset times of each day when caterpillars were L3 were retrieved from [27]. The difference in time of activity and sunset was calculated and rounded to the nearest 15 min because the wildlife camera images were taken every 15 min. The standardised time of activity (minutes before and after sunset) was used as the dependent variable and tested against the independent variables: activity, average daily temperature, colony ID, weather condition and wildlife camera position. Akaike information criterion (AIC) values for all possible models were compared to determine the best model to describe the data. The environmental temperature was not used as an independent variable as it is correlated with the time of activity. An ANOVA was used to assess if the environmental temperature differed from the date of the sampling period (October to November). 

The radii of 100 *T. pityocampa* winter tents were averaged for each cardinal direction: N, E, S and W. The average and SD of the tent radii were plotted using RStudio version 1.2.5033 software package ‘ggplot2′ (Wickham, H., New York, NY, USA). ANOVA was used to assess if there were differences in radii lengths of the final winter tent to determine if the tents were isodiametric or not. 

#### 2.5.3. Polyethism

A total of 988 caterpillars were dissected from the experimental seasons in 2020 and 2021. Individuals with a head capsule width of >2 mm (*N* = 868) were considered as L4 [28], and individuals smaller than this threshold were excluded from the data analyses. The experiment to test polyethism was conducted over 2 years; therefore, we analysed if year influenced the proportion of spinner and forager male and female caterpillars. The total number of spinner and forager male and female caterpillars from 2020 (*N* = 474) were compared against 2021 (*N* = 394) caterpillars using Fisher’s exact test. The proportions of caterpillars from the two years were not significantly different to each other (*p* > 0.5) and were pooled for the following analyses. To determine if more *T. pityocampa* male or female caterpillars were spinners or foragers, sex and activity were analysed using a Chi-square Goodness-of-Fit test for a 50:50 distribution. Additionally, a GLM was used to determine if head capsule widths differed by sex and activity. AIC values for all possible models were compared to determine the best model to describe the data.

## 3. Results

### 3.1. Daily Caterpillar Activity: Frass Production 

Frass production was fairly synchronous with caterpillars feeding on the pine needles captured from the wildlife camera images (Supplementary Figure S4). The frass production by L1 and L2 was relatively continuous throughout the day, although larger fluctuations were observed in L1 compared to L2. It was conspicuous in L1 that more frass was produced during the hours of darkness than in daylight. 

### 3.2. Daily Caterpillar Activity: Tent Construction and Foraging

**Figure 1:**
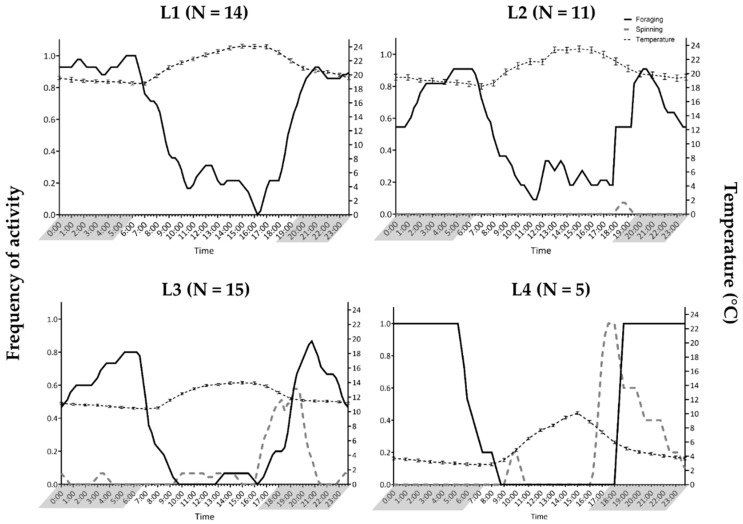
Average daily foraging activity of first to fourth instar and spinning activity of second to third instar *Thaumetopoea pityocampa* caterpillars. The foraging and spinning activity of caterpillars is represented as a solid black line and a grey dashed line, respectively. The average ambient temperature of each hour for each instar has been plotted against the right Y axis. The shaded times on the X-axis represent the hours of darkness.

The spinning behaviour of L1 caterpillars was undetectable from the wildlife camera images because of their small size; therefore, it was analysed from L2 onwards. The colonies from L1 to L4 fed predominantly at night in total darkness (Figure 1). The fluctuations observed during foraging throughout the night are from those colonies that returned to the tent and went back out foraging. Occasionally, some L1 and L2 colonies foraged in the middle of the day. Within L2, only a single colony was involved in spinning. Once the environmental temperature started to drop and when the caterpillars moulted to L3, the silk spinning behaviour was conspicuous (Figure 1). Spinning behaviour in L2 to L4 commenced approximately one to two hours before complete darkness.

**Figure 2:**
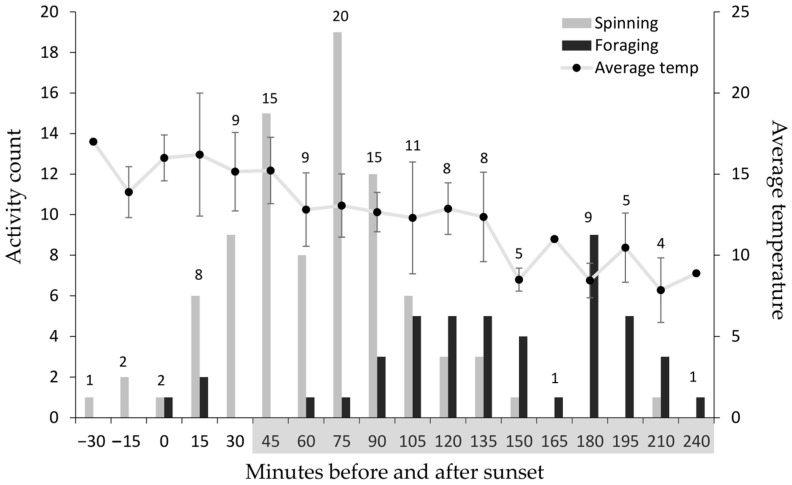
Counts of spinning silk on the tent (grey) and foraging behaviour (black) of third instar (October to November) *Thaumetopoea pityocampa* larval colonies (*N* = 8) plotted against minutes before and after sunset. The Activity count was an accumulative count of the eight L3 colonies recorded from October to November. The shaded grey bar in the minutes before and after sunset indicates darkness in the environment that gradually starts 30 min after sunset. The average temperature (black circle) ± SD (black vertical bar) at the time of activity across the period of observations is displayed on the right Y-axis. The numbers above the bars are the number of observations per minutes before and after sunset.

Thorough tent construction observations were conducted on L3 *T. pityocampa* colonies when they constructed the winter tent. At around sunset, a few early active individuals emerged from the tent and applied silk to the exterior, a process that continued for approximately an hour (Figure 2). After spinning silk, the spinners left the tent and followed the previously laid foraging trails to feed. They were later joined by the rest of the colony that had remained inside the tent. The colony returned from foraging to the tent around sunrise. Tent construction generally occurred when environmental temperatures were >12 °C, whereas foraging generally occurred when it was <12 °C and in darkness (Figure 2). Peak tent construction began 75 min after sunset when there was minimal light, while peak foraging activity began approximately 105 min after peak tent construction behaviour (i.e., 180 min after sunset). The standardised time of when the activity occurred was significantly different from the activity. The time the spinners started constructing the tent was significantly different from the time of foraging (GLM: *t* = −9.19, *N* = 133, *p* = 7.87 × 10^−16^; Figure 2). Other independent variables, such as average daily temperature, colony ID, weather conditions, and wildlife camera position, had no significant effect on the timing of activity (all *p* > 0.1). The environmental temperature was significantly different (ANOVA: *F*_1,131_ = 77.83, *p* = 6.11 × 10^−15^) as the season progressed, with decreasing temperatures from October to November.

**Figure 3:**
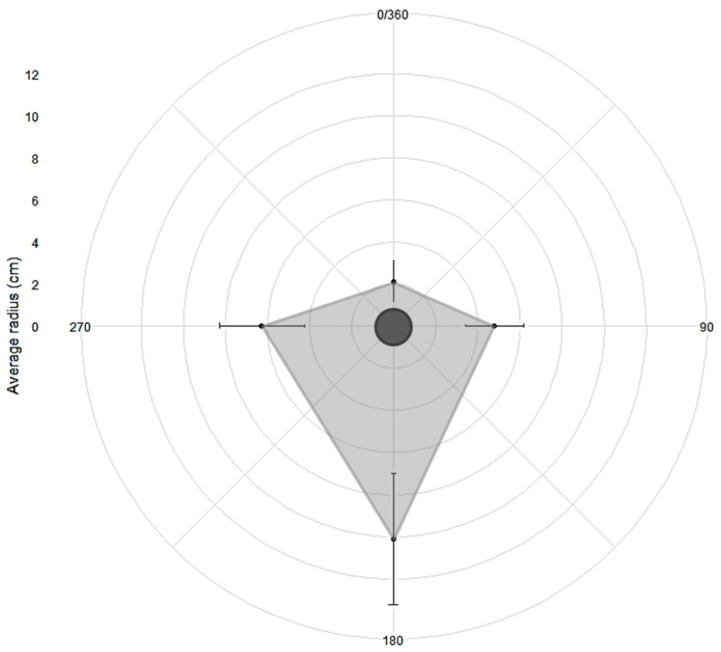
Average tent radii (small black circle) ± SD (black bar) from the pine shoot in the centre (dark grey circle) outwards towards North (0/360), East (90), South (180) and West (270) of final instar *Thaumetopoea pityocampa* winter tents (*N* = 100). Each grey ring represents the distance away from the pine shoot in increments of 2 cm, starting from the shortest distance in the centre to the furthest in the second–last outer ring.

The winter tents constructed by later instar *T. pityocampa* were not isodiametric. Radii lengths between the four compass orientations were significantly different (ANOVA: F_3,396_ = 268.93, *p* < 2.2 × 10^−16^), with a thicker silk layer observed on the southern side (10.1 ± 3.1 cm) of the tent compared to other orientations (N: 2.1 ± 1 cm, E: 4.8 ± 1.4 cm, W: 6.3 ± 2 cm; Figure 3).

### 3.3. Polyethism

**Figure 4:**
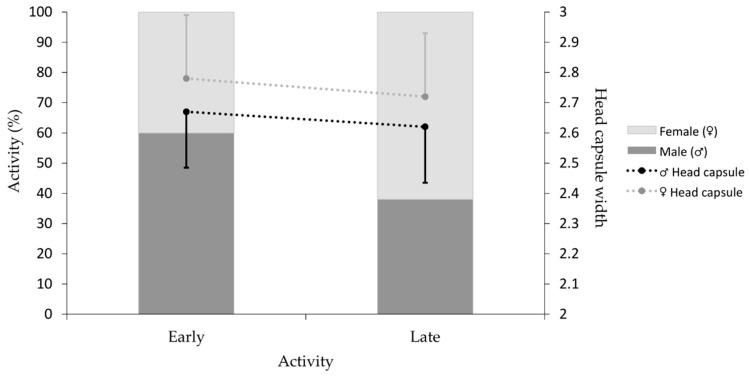
Percentage of early active (caterpillars spinning silk to build the tent and foraging straight after; *N* = 421) and late active (caterpillars foraging from midnight to early morning; *N* = 447) females and males presented in light and dark grey, respectively. Average head capsule widths (±SD) for early and late active males and females are presented as black and grey points, respectively. The standard deviation bars show either positive or negative to prevent the bars from overlapping.

A total of 868 early active (spinners and first foragers, *N* = 421) and late active (late foragers, *N* = 447) L4 *T. pityocampa* caterpillars were dissected from 41 tents to determine the sex of individuals for each activity over time. Of the 868 caterpillars dissected, 51% were female and 49% male. The early active caterpillars were 60% male and 40% female (*N* = 421), whereas the late active caterpillars were 38% male and 62% female (*N* = 447; Figure 4). Both frequencies are significantly different from the expected 50:50 ratio (Early: *X*^2^_1_ = 17.98, *N* = 421, *p* = 2.23 × 10^−5^; Late: *X*^2^_1_ = 26.58, *N* = 447, *p* = 2.53 × 10^−7^). Head capsule measurements of all dissected caterpillars showed that female L4 caterpillars were significantly larger than males irrespective of activity (GLM: *t* = 7.83, *N* = 868, *p* = 1.44 × 10^−14^; Figure 4). Additionally, the head capsule widths of early active caterpillars were significantly larger than late active caterpillars (GLM: *t* = −3.92, *N* = 868, *p* = 9.71 × 10^−5^; Figure 4). These findings suggest that within males, larger individuals were active early in the night, and within females, the smaller individuals emerged later in the night/early morning. 

## 4. Discussion

To understand the tent construction and foraging behaviour of *T. pityocampa*, we needed to examine both behaviours together because they are interconnected. Our findings suggest that *T. pityocampa* caterpillars displayed individual variation through their daily activity of tent construction and foraging. Tent construction was primarily carried out by larger males, while smaller females remained inside the tent and emerged from the tent later to forage in the night. When weather conditions were suitable, early instar *T. pityocampa* caterpillars (L1–2), which are patch-restricted foragers [12], fed at night and occasionally in daylight. Tent construction behaviour in early larval instars was not as evident as in L3 onwards, where spinning silk on the tent becomes crucial due to the drop in environmental temperature. Tent construction by later instar caterpillars occurred when temperatures were above 12 °C at around sunset. After 1–2 h, the caterpillars that were spinning silk began to forage and were joined by the rest of the caterpillars that were inside the tent. Foraging commenced when the environmental temperature dropped below 12 °C, and the environment was in complete darkness. It was common for caterpillars to feed continuously throughout the night and return to the tent before sunrise. On days with heavy rain and wind, the caterpillars did not emerge from the tent for construction or foraging. If the weather improved, the caterpillars resumed their regular foraging pattern once the rain had passed. The final structure of the winter tent constructed by later instar caterpillars had a thicker silk layer in the south, where there was maximum solar radiation compared to the other three cardinal orientations.

Early instar *T. pityocampa* caterpillars fed predominantly at night and occasionally for short periods during the day. In comparison, later instar caterpillars fed exclusively at night in darkness. The short distance the early instar caterpillars travel for food sources [12] and their small size might enable them to forage during the day for brief intervals when predators and parasitoids are active. The relatively warm environmental temperatures experienced by the early instars likely facilitated their digestion and metabolism, as indicated by the frass production that was synchronous with foraging activity. Consequently, this likely enables early instar caterpillars to empty their gut and feed again in a short amount of time. Meanwhile, in later instars (L3–4), the drop in temperature extended the duration of foraging and locomotion to and from the tent and foraging sites, as described by [9]. Later instar *T. pityocampa* caterpillars are central place foragers [12] and follow previously laid foraging trails in a procession to pine needles further away from the tent. Their larger size, which makes them conspicuous, and the longer distance to travel to food sources may have influenced their nocturnal foraging behaviour for parasitoid and predator evasion.

Later instar caterpillars commenced foraging when the temperature dropped below 12 °C, and it was completely dark. This drop in temperature and complete darkness may be a stimulus for caterpillars to cease tent construction and initiate foraging. Future studies should manipulate environmental temperature and visible light to determine if they affect the timing of tent construction and foraging in *T. pityocampa* colonies. In our study using wildlife camera timelapse images, we observed two groups of caterpillar activity instead of the three described by [9]. Therefore, continuous macro videography of L3 tent construction behaviour is required to determine if the three activities described by [9] can be detected. 

Until this study, the comparison of silk application on the winter tent of the four cardinal orientations has not been measured for *T. pityocampa*. The south was the preferred orientation for *T. pityocampa* caterpillars to apply silk on the winter tent. Multiple studies on *T. pityocampa* have shown southerly facing winter tent orientation on the host tree [12,23,29]. Larval preference for the south for tent orientation and silk application on the winter tent clearly demonstrates that it is to receive maximum solar radiation. Preference for tent building on the southerly orientation of the tree and spinning silk on the most intensely illuminated side of the tent has also been demonstrated in *M. americanum* caterpillars [5]. Maximum insolation is necessary, especially for later instar *T. pityocampa* caterpillars when they must endure winter conditions. Tent construction/maintenance behaviour commenced at sunset when the polarised band was the strongest along the zenith [30]. This suggests that *T. pityocampa* caterpillars can determine the southern-facing side of the tent by using the position of the sun and skylight polarisation pattern at sunset for silk application on the tent, similar to how they orientate on the ground during the pre-pupation procession (see [31]). 

At sunset, not all *T. pityocampa* caterpillars emerged from the tent to spin silk on the tent structure. From the dissections, we determined that the early active caterpillars constructing the tent were larger individuals, primarily males and some females. In comparison, the later active individuals that emerged from the tent in the evening/early morning to forage were smaller, mainly females and some males. This finding opens an extensive discussion about the possible explanations for the involvement of larger individuals and primarily male caterpillars in tent construction. *Thaumetopoea pityocampa* caterpillar spins and attaches silk by arching the anterior part of its body backwards to a substrate [9]. Therefore, larger individuals spinning silk for tent construction may be advantageous as they can cover a larger surface area or perhaps have a larger silk gland reservoir. Silk production is a costly investment, accounting for 18% of ingested nitrogen in parsnip webworms, *Depressaria pastinacella* [32]. Consequently, individuals with higher nitrogen levels may focus on tent construction, while individuals with lower levels may only forage. More male caterpillars constructing the tent than females may indicate polyethism in *T. pityocampa* as described in *Eucheira socialis*. In *E. socialis*, the male caterpillars were active first in the colony, spent more time spinning silk on the nest and were first to embark on foraging forays [10,11,33]. It was suggested that male *E. socialis* take on the role of ‘workers’ [33], thereby enabling female caterpillars to conserve resources for later use in egg production [11]. Apart from tent construction, the final instar *T. pityocampa* caterpillar processions that leave the tent permanently to find a pupation site are predominantly led by a single female leader [34]. This may suggest that both male and female *T. pityocampa* caterpillars have different responsibilities within the colony. It is clear that multiple variables exist, and research remains to be performed to determine if *T. pityocampa* individuals have different tasks within the colony based on body size and sex. 

The stimuli that trigger the change of behaviour from remaining inside the tent to spinning silk on the tent to foraging in social caterpillars remain unknown and require further exploration. The combination of task preference and response thresholds to stimuli may generate polyethism within a colony [33,35,36]. At the onset of darkness, male caterpillars may be more sensitive to environmental cues, prompting them to initiate tent construction tasks and emerge from the nest earlier than females [33]. Female caterpillars that emerge later may lack silk-spinning stimuli and immediately engage in foraging [11]. In highly social caterpillars, effective cooperation and communication among individuals within the colony are essential for successful tent construction [37]. *Malacosoma americanum* caterpillars engage in tent construction at specific times to delay the departure of the entire colony from emerging and allow time for individuals to assemble on the tent surface [5]. Coordinated group assembly and spinning may be essential for tent expansion and foraging efficiency [5]. Unlike *M. americanum* tents, which have one exit hole [5], in *T. pityocampa* tents, the caterpillars can exit from any direction within the tent (Uemura, pers. obs.). Limiting the number of caterpillars being stimulated to spin silk and emerging from the tent all at once could help retain the integrity of the tent structure. Furthermore, having a few individuals respond to the stimuli for tent construction ensures spinners can lay down the silk efficiently without overcrowding. Additionally, with most individuals inside the tent and only some spinning on the outside of the tent, it reduces the likelihood of the colony to encounter natural enemies. Determining if the same individuals are consistently a spinner or forager will be required if polyethism exists in *T. pityocampa*. Marking spinners and foragers with different coloured paint, tracking them automatically through video tracking tagged individuals, and investigating the fatality of spinners vs. foragers from natural enemies could provide insights into task allocation in *T. pityocampa* colonies. 

## 5. Conclusions

Similarly to various other insect shelters, the *T. pityocampa* winter tent offers protection against environmental elements and natural enemies but also facilitates thermoregulation [18,20,21]. The importance of the winter tent for *T. pityocampa* was further demonstrated in our study by the synchronised and consistent timing of daily tent construction by the spinners. Investigating tent construction and foraging behaviour of *T. pityocampa* colonies contributed additional insights to the collective nest building and social behaviours observed in Lepidoptera and non-eusocial insects. The initiation of tent construction, primarily undertaken by larger male *T. pityocampa* caterpillars, opens up a broader discussion on polyethism and the roles of individuals in non-eusocial insect colonies. It will be important to address performance, mortality and the costs and benefits of behavioural task differences in *T. pityocampa* colonies. In this study, the use of wildlife cameras and the novel frass counting apparatus demonstrated non-destructive field collection methods that capture the natural behaviour of these insects. This approach creates opportunities for researchers to monitor insects in the field over extended periods without causing disturbance. 

## Data Availability

The data generated and analysed for this study is available from the University of Padova repository: https://researchdata.cab.unipd.it/id/eprint/789 (accessed on 6 December 2022).

## Supplementary Materials

The following supporting information can be downloaded at: https://www.mdpi.com/article/10.3390/insects14100829/s1, Figure S1: Satellite image of the *Pinus nigra* stand study site in Precastio, Verona, Italy, where *Thaumetopoea pityocampa* colonies were monitored for tent construction and foraging behaviour. Figure S2A–D: Experimental set-up of the frass collecting experiment on young instar caterpillars in the Precastio study site. Function S1: Methods on how to use ImageJ to count frass from an image digitally. Figure S3: Successive photographs of one *T. pityocampa* tent from the third (L3) to final (L5) instar larva. Figure S4: Average daily feeding activity and frass production of first (L1, *N* = 14 and 7) and second (L2, *N* = 11 and 5) instar *Thaumetopoea pityocampa* caterpillars.

## References

[1] Reader T., Hochuli D.F. (2003). Understanding Gregariousness in a Larval Lepidopteran: The Roles of Host Plant, Predation, and Microclimate. Ecol. Entomol..

[2] Theraulaz G., Bonabeau E., Deneubourg J.L. (1998). The Origin of Nest Complexity in Social Insects. Complexity.

[3] Fitzgerald T.D., Costa J.T., Detrain C., Deneubourg J.L., Pasteels J.M. (1999). Collective Behavior in Social Caterpillars. Information Processing in Social Insects.

[4] Snodgrass R.E. (1921). The Fall Webworm. Ann. Rep. Smithsonian Inst..

[5] Fitzgerald T.D., Willer D.E. (1983). Tent-Building Behavior of the Eastern Tent Caterpillar *Malacosoma americanum* (Lepidoptera: Lasiocampidae). J. Kansas Entomol. Soc..

[6] Ruf C., Freese A., Fiedler K. (2003). Larval Sociality in Three Species of Central-Place Foraging Lappet Moths (Lepidoptera: Lasiocampidae): A Comparative Survey. Zool. Anz..

[7] Dussutour A., Nicolis S.C., Despland E., Simpson S.J. (2008). Individual Differences Influence Collective Behaviour in Social Caterpillars. Anim. Behav..

[8] Mills M. (1951). Bag Shelter Caterpillars and Their Habits. West. Aust. Nat..

[9] Démolin G. (1967). Grégarisme et Subsocialité Chez *Thaumetopoea pityocampa* Schiff. Nid d’hiver—Activité de Tissage. Proceedings of the C. R. Ve Congrès de L’union Internationale Pour L’étude des Insectes Sociaux.

[10] Underwood D.L.A., Shapiro A.M. (1999). A Male-Biased Primary Sex Ratio and Larval Mortality in *Eucheira socialis* (Lepidoptera: Pieridae). Evol. Ecol. Res..

[11] Underwood D.L.A., Shapiro A.M. (1999). Evidence for Division of Labor in the Social Caterpillar *Eucheira socialis* (Lepidoptera: Pieridae). Behav. Ecol. Sociobiol..

[12] Uemura M., Zalucki M.P., Battisti A. (2020). Behavioural Plasticity and Tree Architecture Shapes Tent and Foraging Locations of Pine Processionary Larval Colonies. Entomol. Gen..

[13] Battisti A., Avcı M., Avtzis D.N., Ben Jamaa M.L., Berardi L., Berretima W., Branco M., Chakali G., El Alaoui El Fels M.A., Frérot B., Roques A. (2015). Natural History of the Processionary Moths (*Thaumetopoea* spp.): New Insights in Relation to Climate Change. Processionary Moths and Climate Change: An Update.

[14] Battisti A., Holm G., Fagrell B., Larsson S. (2011). Urticating Hairs in Arthropods: Their Nature and Medical Significance. Annu. Rev. Entomol..

[15] Ferchault de Réaumur R.-A. (1734). Memoires Pour Servir a L’histoire des Insectes.

[16] Fabre J. (1898). Souvenirs Entomologiques. Sér.

[17] Battisti A., Stastny M., Netherer S., Robinet C., Schopf A., Roques A., Larsson S. (2005). Expansion of Geographic Range in the Pine Processionary Moth Caused by Increased Winter Temperatures. Ecol. Appl..

[18] Poitou L., Robinet C., Suppo C., Rousselet J., Laparie M., Pincebourde S. (2021). When Insect Pests Build Their Own Thermal Niche: The Hot Nest of the Pine Processionary Moth. J. Therm. Biol..

[19] Battisti A., Hódar J.A., Hernández R., Larsson S. (2022). Aggregative Oviposition Varies with Density in Processionary Moths — Implications for Insect Outbreak Propensity. Ecol. Entomol..

[20] Hódar J.A., Castro J., Zamora R. (2003). Pine Processionary Caterpillar *Thaumetopoea pityocampa* as a New Threat for Relict Mediterranean Scots Pine Forests under Climatic Warming. Biol. Conserv..

[21] Branco M., Santos M., Calvão T., Telfer G., Paiva M. (2008). Arthropod Diversity Sheltered in *Thaumetopoea pityocampa* (Lepidoptera: Notodontidae) Larval Nests. Insect Conserv. Divers..

[22] Uemura M., Perkins L.E., Zalucki M.P., Battisti A. (2020). Movement Behaviour of Two Social Urticating Caterpillars in Opposite Hemispheres. Mov. Ecol..

[23] Breuer M., Devkota B., Douma-Petridou E., Koutsaftikis A., Schmidt G.H. (1989). Studies on the Exposition and Temperature of Nests of *Thaumetopoea pityocampa* (Den. & Schiff.) (Lep., Thaumetopoeidae) in Greece. J. Appl. Entomol..

[24] Lavenseau L. (1982). Determination of the Sex of Caterpillars without Dissection. Int. J. Insect Morphol. Embryol.

[25] Underwood D.L.A. (1994). Methods for Sexing Lepidoptera Larvae Using External Morphology. J. Lepid. Soc..

[26] Battisti A. (1988). Phytophagous Insects in the Energy Flow of an Artificial Stand of Pinus Nigra Arnold in Northern Italy. Redia.

[27] Hoffman T. SunCalc. www.suncalc.org/.

[28] Grison P., Sylvestre R.d.S., Galichet P.F. (1951). La Processionnaire Du Pin (*Thaumetopoea pityocampa* Schiff.). Mœurs, Dégâts, Moyens de Lutte. Rev. Zool. Agric. Appl. Talence.

[29] Sebti S., Chakali G. (2014). Distribution and Importance of the Pine Processionary Moth Winter Nests *Thaumetopoea pityocampa* (Denis & Schiffermüller) (Lepidoptera: Notodontidae) in the Forests Cedar of the National Park of Chréa (Algeria). Int. J. Agric. Sci. Res..

[30] Cronin T.W., Warrant E.J., Greiner B. (2006). Celestial Polarization Patterns during Twilight. Appl. Opt..

[31] Uemura M., Meglič A., Zalucki M.P., Battisti A., Belušič G. (2021). Spatial Orientation of Social Caterpillars Is Influenced by Polarized Light. Biol. Lett..

[32] Berenbaum M.R., Green E.S., Zangerl A.R. (1993). Web Costs and Web Defense in the Parsnip Webworm (Lepidoptera: Oecophoridae). Environ. Entomol..

[33] Yayalar N.N. (2009). The Influence of Sex-Specific Behaviors on Individual Growth and Mortality of a Caterpillar, Eucheira Socialis Westwoodi.

[34] Démolin G. (1971). Incidences de Quelques Facteurs Agissant sur le Comportement Social des Chenillesde *Thaumetopoea pityocampa* Schiff. (Lepidoptera) Pendant la Période des Processions de Nymphose. Répercussion sur l’efficacité des Parasites. Ann. Zool. Ecol. Anim..

[35] Despland E., Hamzeh S. (2004). Ontogenetic Changes in Social Behaviour in the Forest Tent Caterpillar, *Malacosoma disstria*. Behav. Ecol. Sociobiol..

[36] Nicolis S.C., Despland E., Dussutour A. (2008). Collective Decision-Making and Behavioral Polymorphism in Group Living Organisms. J. Theor. Biol..

[37] Ruf C., Fiedler K. (1999). Colony Survivorship of Social Caterpillars in the Field: A Case Study of the Small Eggar Moth (Lepidoptera: Lasiocampidae). J. Res. Lepid..

