# Supplementary material for: Large Male Caterpillars Are the Primary Builders: Exploring Tent Construction and Foraging Behaviour in Gregarious Pine Processionary Caterpillar
Source: Insects. 2023 Oct 21;14(10):829. doi: 10.3390/insects14100829 (PMC10607817; doi:10.3390/insects14100829)
Supplement: Supplementary file 1 [file insects-14-00829-s001.zip › insects-2643340-supplementary.pdf]

Supplementary materials

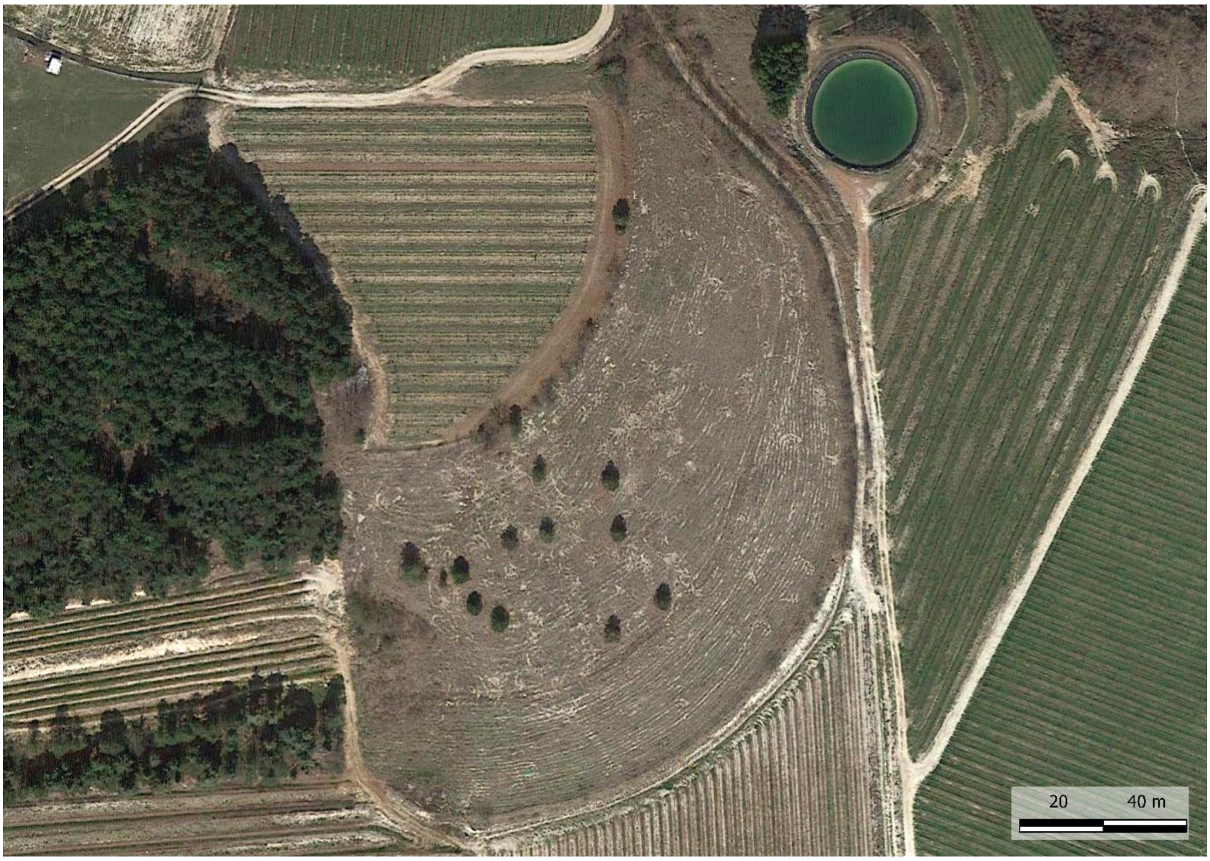

Figure S1. *Pinus nigra* stand in Precastio, Verona Italy, a study site where *Thaumetopoea pityocampa* colonies were monitored for foraging and tent construction behaviour. Satellite image was taken on 17/03/2020 by Google Earth Pro, image accessed 18/05/2021.

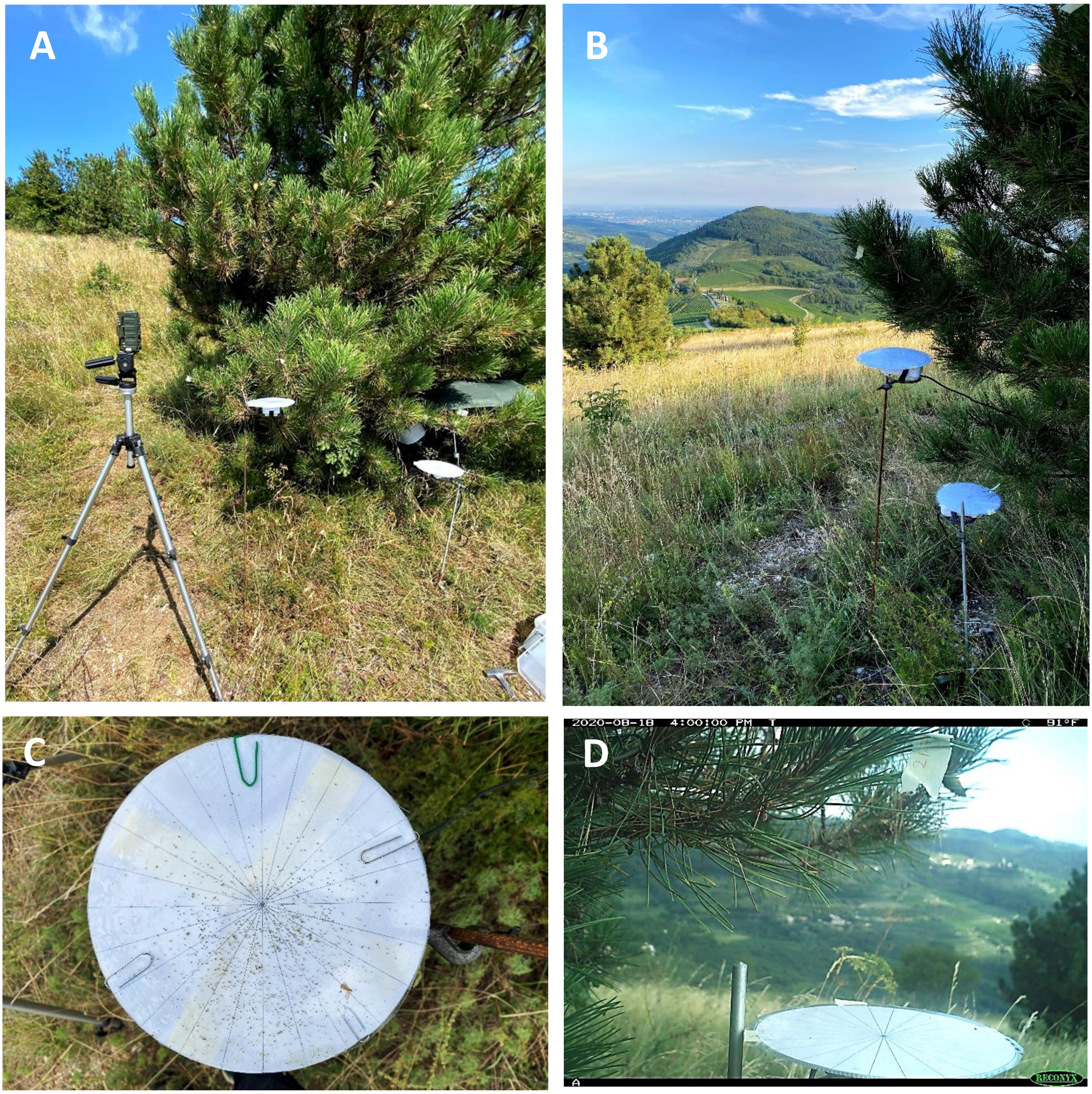

Figure S2. (A,B) Experimental set up for counting frass from *T. pityocampa* colonies in Precastio, Verona Italy. Rotating discs were positioned directly underneath a second larval instar (L2) colony. (**C**) Image of the rotating disc after 24 h of collecting frass from a L2 colony. (**D**) Image taken from the wildlife camera showing the rotating disc underneath a L1 colony.

Supplementary Material Functions S1

1. Open a cropped image (one slice of 24 slices) in imageJ and remove big objects (e.g. pine needle, fallen *T. pityocampa* larvae, etc.) that are not frass by circling the image using any of the shapes in the toolbar → click Edit → click Fill

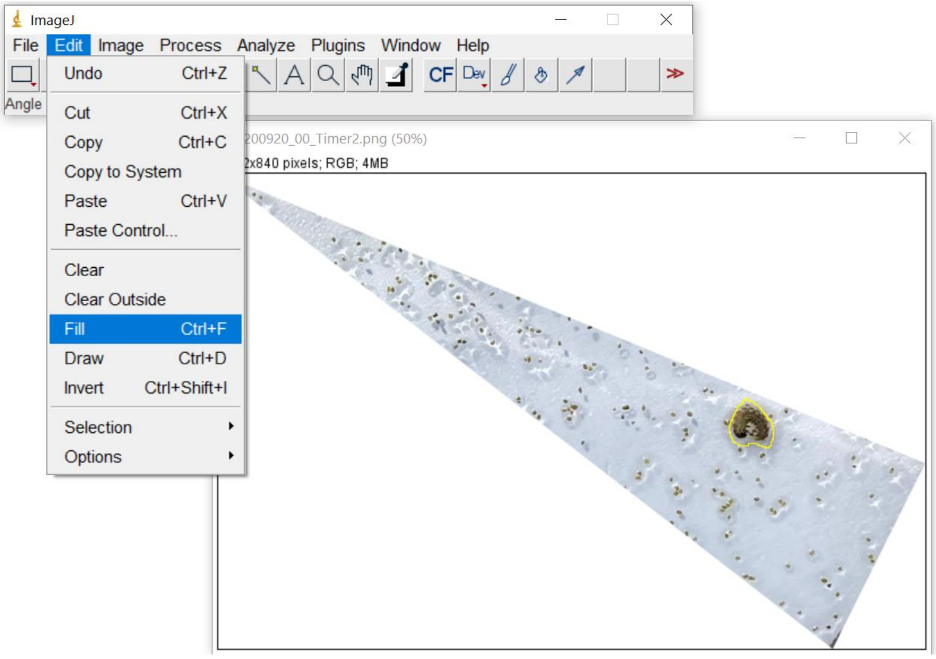

2. Format image to easily view the frass (particles) by clicking: Process → Binary → Make Binary

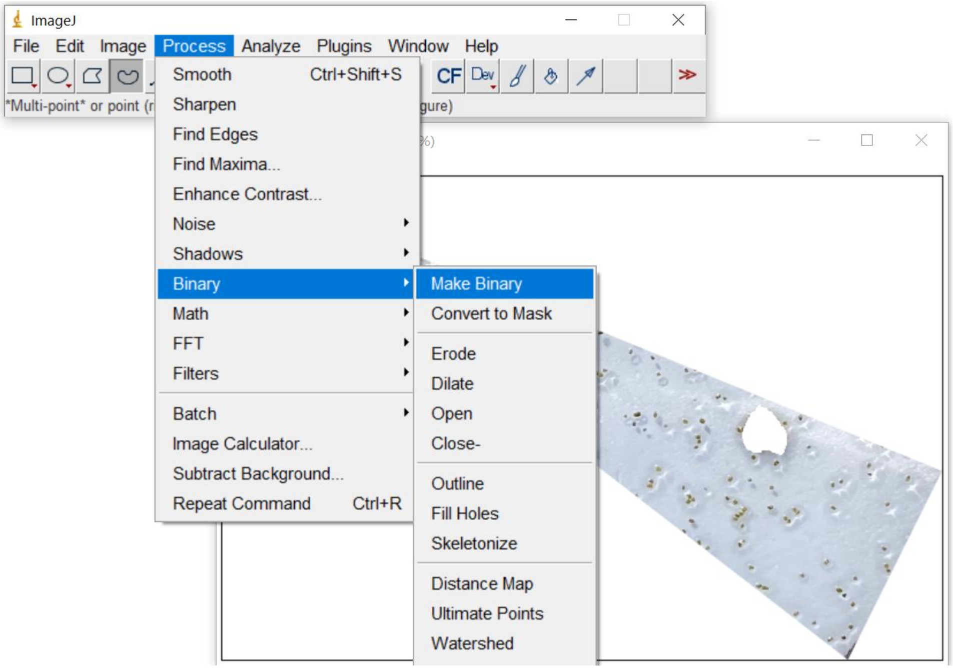

3. Count caterpillar frass in the image by clicking Analyze → Analyze particles → In the pop-up window set “Show:” to Outlines → tick Summarize and In situ Show → OK

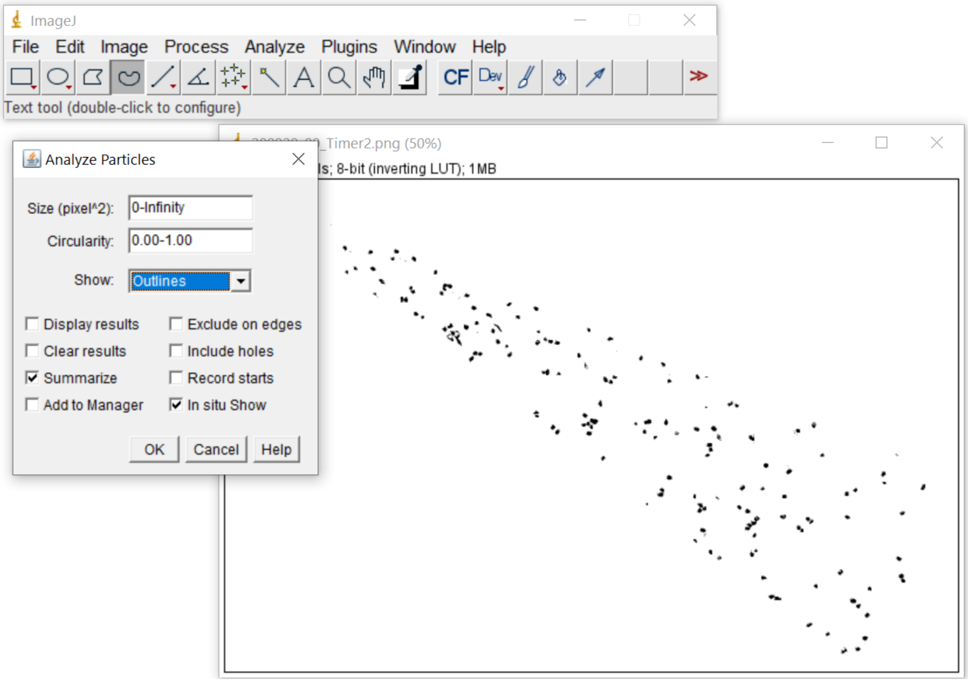

4. Continue this for each slice/cropped image (24 slices in total for one image) from the same image and the Summary table will accumulate with all the results → Press Ctrl + A → Press Ctrl + C to copy all data in the Summary Table and input in Excel file. The ‘Count’ is the number of frass (particles) that were counted in the image.

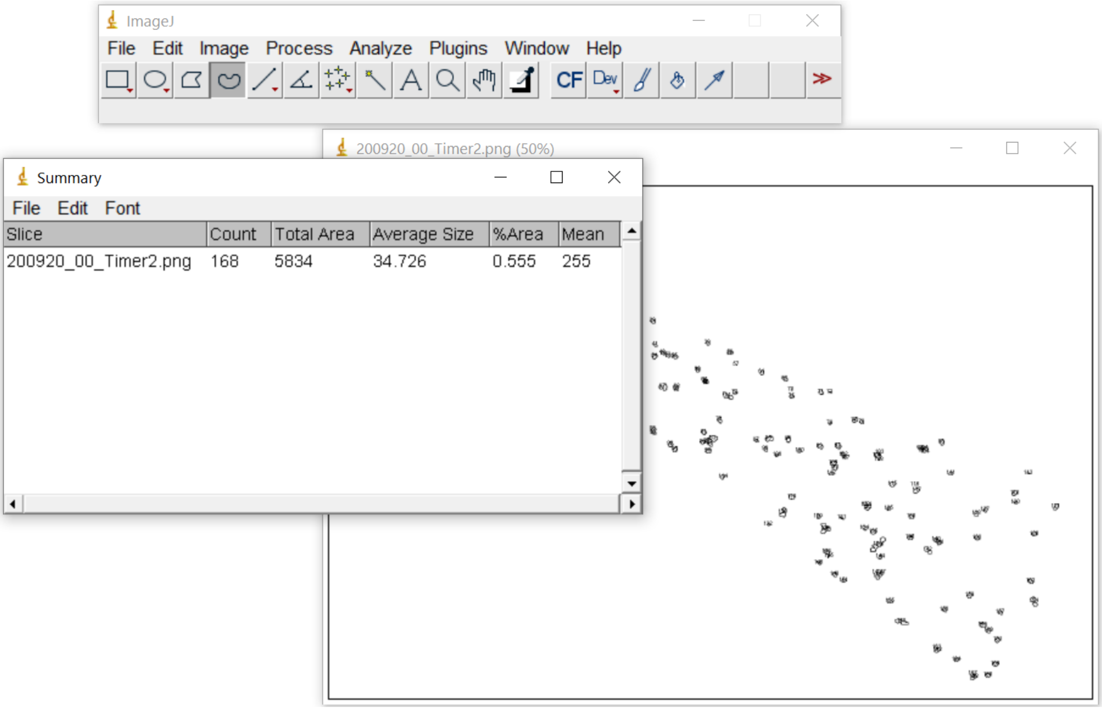

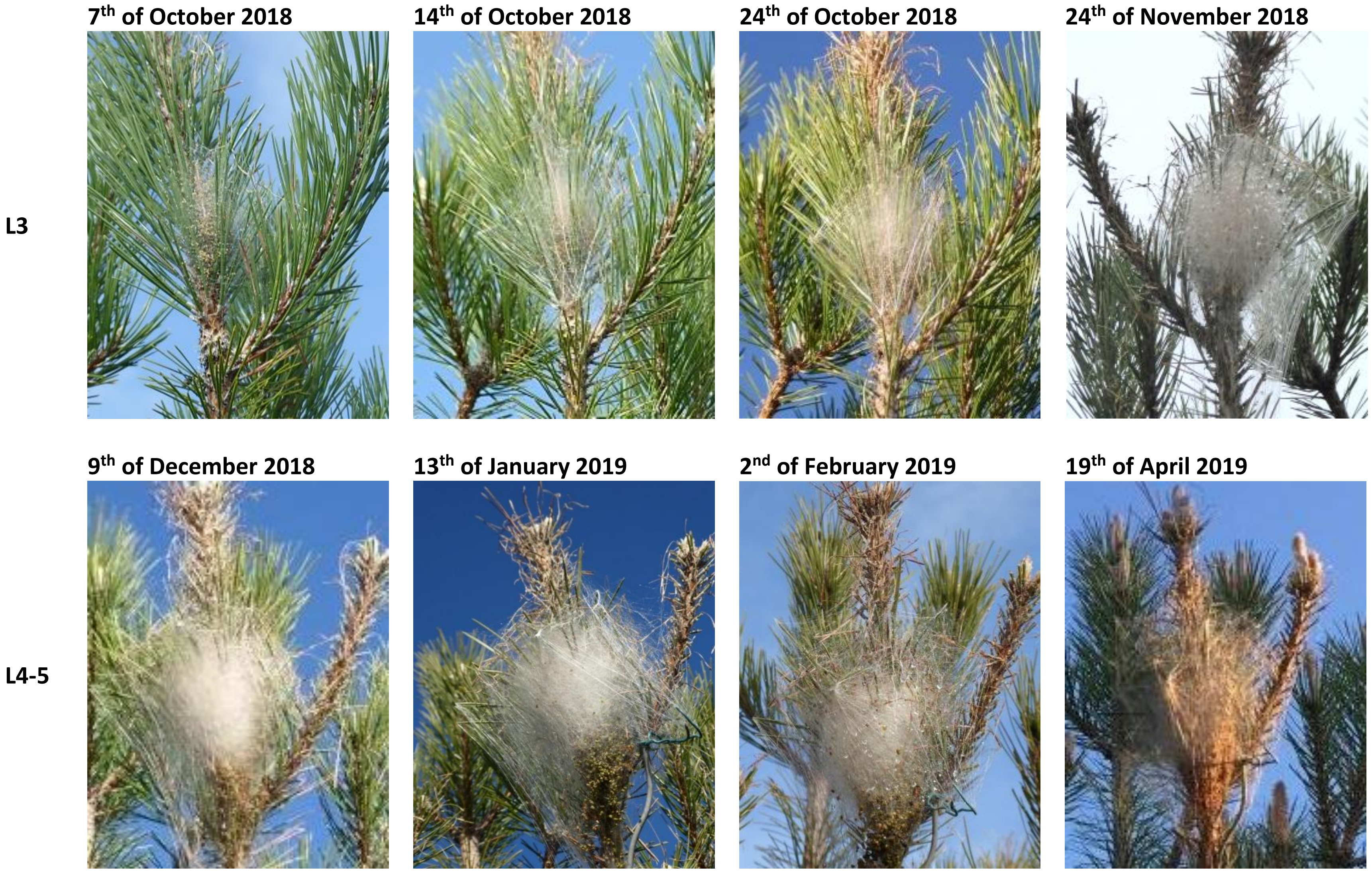

Figure S3. Successive photographs of one *T. pityocampa* tent from third (L3) to final (L5) instar larva. Photographs taken by Andrea Battisti.

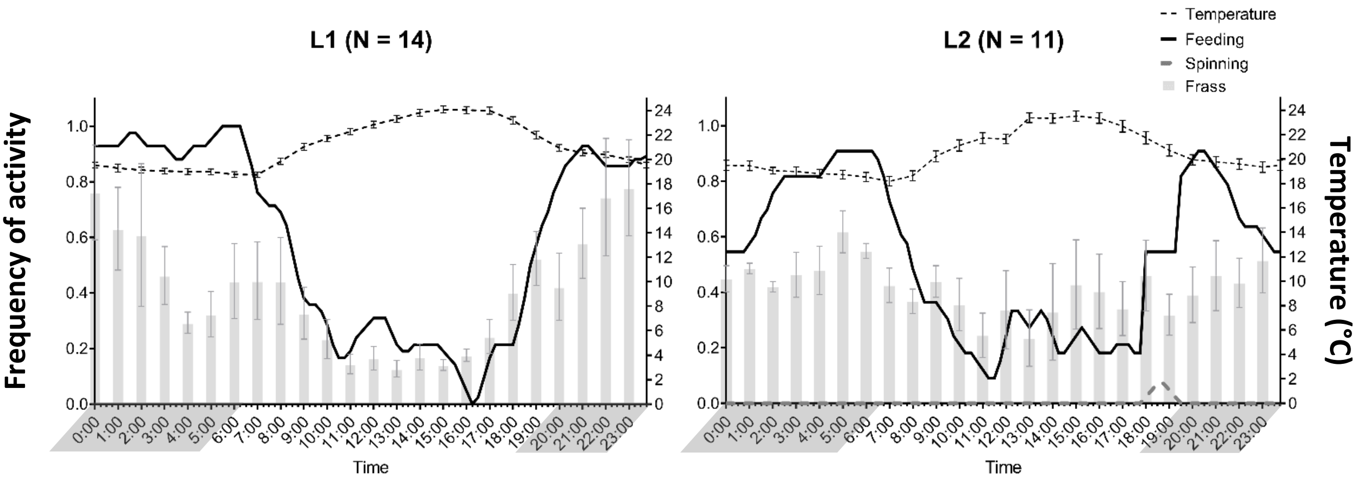

Figure S4. Average daily feeding activity and frass production of 1st (L1, N = 14 and 7) and 2nd (L2, N = 11 and 5) instar *Thaumetopoea pityocampa* caterpillars. The feeding and spinning activity of caterpillars is represented as a solid black line and grey dashed line, respectively and the frass production is represented as grey box plots. The two activities and frass production have been averaged, standardised, and plotted against the left Y axis. For graphical reasons, the proportion of frass production has been altered to the relative scale by 10 times. The average ambient temperature of each hour for each instar has been plotted against the right Y axis. The shaded times on the X axis represents the hours of darkness.
